# Novel 3D Printing Phase Change Aggregate Concrete: Mechanical and Thermal Properties Analysis

**DOI:** 10.3390/ma15238393

**Published:** 2022-11-25

**Authors:** Jinyang Jiang, Chaolang Zheng, Fengjuan Wang, Wenxiang Xu, Liguo Wang, Zhaoyi Chen, Wei Su

**Affiliations:** 1 School of Material Science and Engineering, Southeast University, Nanjing 211189, China; 2 College of Mechanics and Materials, Hohai University, Nanjing 211100, China; 3 China Railway Design Corporation, Binhai New Area, Tianjin 300308, China

**Keywords:** phase change materials, 3D printing, ductility, thermal properties, latent heat

## Abstract

The use of phase change materials (PCMs) in concrete is a double-edged sword that improves the thermal inertia but degrades the mechanical properties of concrete. It has been an essential but unsolved issue to enhance the thermal capacity of PCMs while non-decreasing their mechanical strength. To this end, this work designs a novel 3D printing phase change aggregate to prepare concrete with prominent thermal capacity and ductility. The work investigated the effects of 3D printing phase change aggregate on the compressive strength and splitting tensile strength of concrete. The compressive strength of phase change aggregate concrete is 21.18 MPa, but the ductility of concrete improves. The splitting tensile strength was 1.45 MPa. The peak strain is 11.69 × 10^−3^, nearly 13 times that of basalt aggregate concrete. Moreover, using 3D printing phase change aggregate reduced concrete’s early peak hydration temperature by 7.1%. The thermal insulation capacity of the experiment cube model with phase change concrete has been improved. The results show that the novel 3D printing change aggregate concrete has good mechanical properties and latent heat storage, providing a guideline for applying PCMs in building materials.

## 1. Introduction

The development of human society is inseparable from energy. With the growth of the world population and the improvement of living standards, energy consumption in human society has increased steadily. In developed countries, building energy consumption accounts for 20–40% of the total energy consumption, and the energy consumption for HVAC (heating, ventilation, air-conditioning, and cooling) systems can reach 50% [1,2,3]. Phase change material (PCM) has the characteristics of high energy storage density, high latent heat, and stable temperature in the phase change process. It can reversibly store a large amount of heat at a constant working temperature and re-release it at an appropriate time to realize the rational redistribution of energy supply and demand in time and space and significantly reduce the energy consumption of buildings [4,5]. Developing phase change materials and studying phase change energy storage are practical solutions to reduce building energy consumption.

The combination of phase change materials and civil engineering materials is an important research field [6]. There have been a lot of studies on how to apply phase change materials to civil engineering materials. The combination methods of phase change materials and civil engineering materials are mainly divided into four categories: direct incorporation, immersion, shape-stabilization, and encapsulation [7]. Direct incorporation is very similar to immersion. The difference is that the direct incorporation method is to add PCM during the mixing and preparation of building materials such as gypsum, mortar, or concrete, while immersion technology is to immerse building materials into liquid PCM after the preparation and absorb PMC through capillary action. These two methods have the advantages of simplicity and low cost. Still, they have some disadvantages, such as the loss of phase change materials, affecting hydration products, reducing the bond strength between aggregate and cementitious materials, and reducing concrete’s mechanical properties and durability [8,9,10,11,12]. Shape-stabilization technology can make up for the shortcomings of the above two methods to a certain extent. It can significantly reduce the leakage of phase change materials in the phase change process and increase the thermal stability of phase change materials under multiple thermal cycles. In the shape-stabilization technology, the supporting materials such as high-density polyethylene (HDPE), polystyrene, porous metal foam, and porous silica are fully mixed with the melted PCM, then cooled until solidification so that PCM is formed in the nanovoid, forming a non-melting layer, which greatly improves the thermal stability of phase change materials [13,14,15,16,17,18]. However, the support material absorbing PCM contains a large number of voids, and the strength of the phase change composite is low, which will still reduce the mechanical properties of concrete [19]. Encapsulation further isolates the direct contact between PCM and civil engineering materials, and there are more combinations of shell and core, so it has higher designability. Encapsulation includes microencapsulation and macro encapsulation [20]. Microencapsulation technology uses physical or chemical methods to disperse PCM and then wrap it in a thin shell. This method’s advantage is improving heat transfer efficiency through its large specific surface area. The disadvantage is that the preparation of phase change microcapsules is difficult, the preparation process is complex, and the strength of the product is low and easy to damage [21,22,23,24]. V.D. Cao [25] added phase change microcapsules into Portland cement concrete (PCC) and geopolymer concrete (GPC), respectively. With the increase of the amount of phase change microcapsules, the compressive strength decreased significantly; Through scanning electron microscopy, many researchers observed that microcapsules would break in building materials [26,27,28]. Macro-encapsulation technology puts phase change materials into large containers of spherical, tubular, plate, or other shapes. This method has the advantages of large reserves of phase change materials, easy transportation and treatment, and is suitable for prefabricated components. The disadvantage is that the protective shell wrapped in the outer layer of PCM will reduce the thermal conductivity and heat transfer efficiency [29,30,31,32,33,34,35].

Therefore, given the disadvantages of poor thermal stability and reduced mechanical properties of phase change materials used in building materials, this paper is committed to proposing a new macro-encapsulated phase change concrete aggregate. Combining PCM with concrete aggregate realizes the function of phase change energy storage without significantly reducing the mechanical properties of concrete. Based on bionic technology and imitating the shape of a virus, the concrete aggregate with an external tentacle and the internal cavity is designed and prepared by 3D printing. Paraffin, as phase change material, is encapsulated in the internal cavity of 3D printed aggregate. The strength of phase change aggregates mortar, and the strength of continuously graded phase change aggregate concrete are studied, respectively. The effect of reducing hydration heat and heat preservation and temperature regulation function of phase change aggregate concrete were studied.

## 2. Materials and Methods

### 2.1. 3D Printing Aggregate Preparation

**Figure 1:**
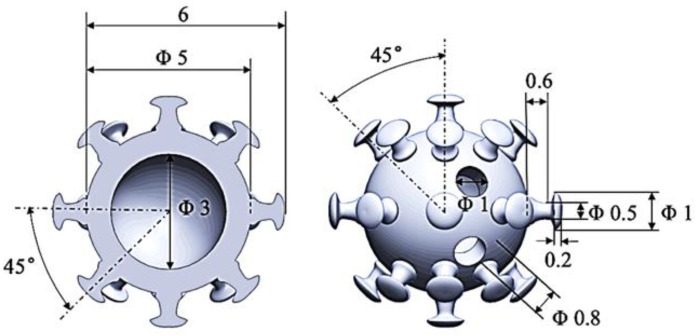
3D printing phase change aggregate structure design.

The spherical structure was selected as the basic configuration of phase change aggregate to reduce the adverse effects of the aggregate shape on the workability and mechanical properties of concrete. Based on bionic technology, imitating the structure of the bacteria and viruses, a tentacle-like secondary structure was designed on the surface of the sphere to increase the contact area. A cavity was set inside the spherical structure to accommodate the PCMs, and two small holes were reserved on the surface to facilitate the injection of PCMs. The detailed structural information of phase change aggregate is shown in Figure 1.

**Figure 2:**
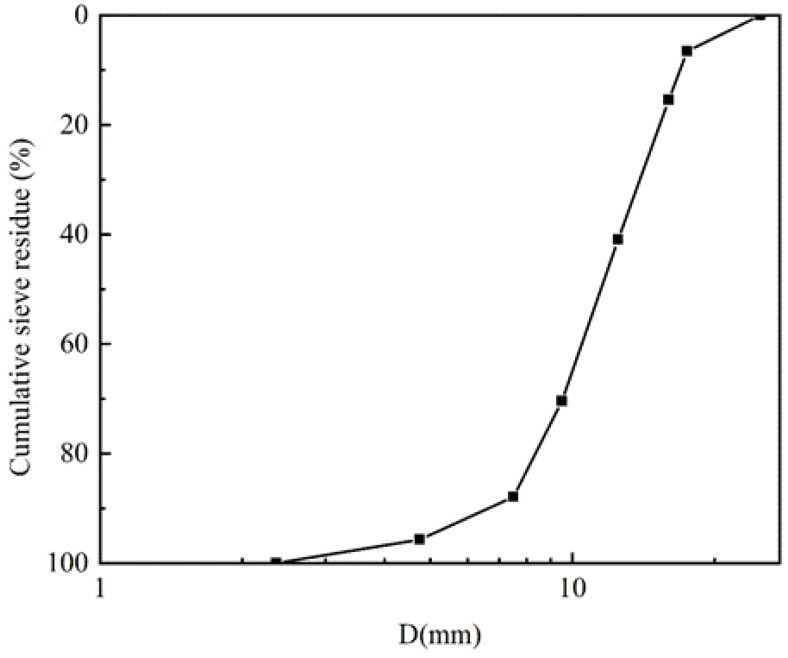
Grading curve of phase change aggregate. (The coordinates of the small squares indicate the cumulative sieve residue of the phase change aggregates at the corresponding size).

**Figure 3:**
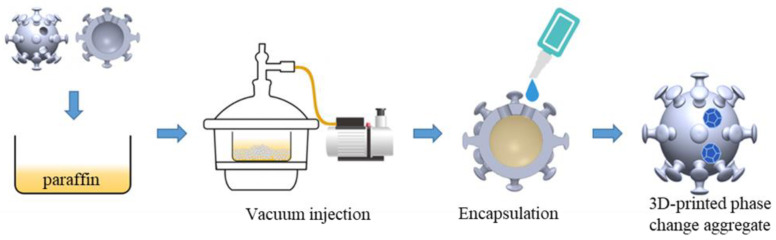
Encapsulation process of phase change aggregate. (The 3D-printed aggregates are vacuum impregnated in molten paraffin wax and then encapsulated with epoxy resin in the small holes reserved for the surface.).

Good gradation can make the aggregate stack more compact and enhance the mechanical properties of concrete. The phase change aggregate of each particle size is shown in Table 1, and the gradation curve is shown in Figure 2:

A JennyLight 1 + LCD light curing 3D printer prepared the phase change aggregate. The prepared parameters include the bottom exposure time of 30 s, the bottom layer number 5, and the common layer exposure time of 10 s. The printing materials are bisphenol F epoxy acrylate resin, TPGDA as the diluent, and TPO as the light initiator. After printing, it was cleaned with anhydrous ethanol, put into the curing box, and irradiated under a purple lamp for 30 min to improve the strength. Finally, the melted paraffin was filled with the cavity of the phase change aggregate, and the holes reserved on the surface were sealed with epoxy resin glue. The encapsulation process is shown in Figure 3.

### 2.2. Other Raw Materials

The cement used in the test was P·II 52.5 cement produced by Nanjing Jiangnan Onoda Cement Co., Ltd., Nanjing, China. and its chemical composition is shown in Table 2. The chemical composition of the fly ash used is shown in Table 3. The sand was used with an apparent density of 2600 kg/m^3^, fineness modulus of 2.4, and water absorption rate of 1.0%. 5–20 mm continuous graded basalt gravel was used as coarse aggregate. Polycarboxylic acid series superplasticizer with a solid content of 39.9% was used.

### 2.3. Sample Preparation

The cement-based material system of cement and fly ash is adopted with a mass-based water-to-cement ratio (*w/c*) = 0.4, as shown in Table 4. In addition to the control M−0 mortar, phase change mortars add basalt aggregate and phase change aggregate filled with different volume fraction PCM. The cavity of phase change aggregate included PCMs at four volumetric inclusion levels of 0%, 33%, 66%, and 99%. In the early stages of the experiments, 3D printing of phase change aggregates was very inefficient, and the use of reduced size non-standard size samples greatly accelerated the progress of the experiments. We prepared the size of the cementitious composites as 40 mm × 40 mm × 40 mm non-standard samples. We used non-standard samples to investigate the effect of phase change aggregates on the mechanical properties of this cementitious composite. Their strength was only compared with samples of the same size. Limited by the sample geometry size, each sample contains six coarse aggregates (a 16 mm aggregate, a 12.5 mm aggregate, and four 4.75 mm aggregates). To research the effect of volume fraction of PCM on compressive strength. The phase change aggregate filled with PCM was used to replace the basalt aggregates with 0%, 50%, and 100% by volume, respectively, as shown in Table 5. 100 mm × 100 mm × 100 mm samples were prepared. After storing in a moist chamber (>95% RH, 20 ± 2 °C) until 28 d of testing, the compressive and splitting properties were tested to study the effect of phase change aggregate on the mechanical properties of concrete. The 300 mm × 300 mm × 30 mm concrete slab was prepared with phase change aggregate and coarse basalt aggregate, respectively, to study its heat preservation and temperature regulating performance.

### 2.4. Test Methods

**Figure 4:**
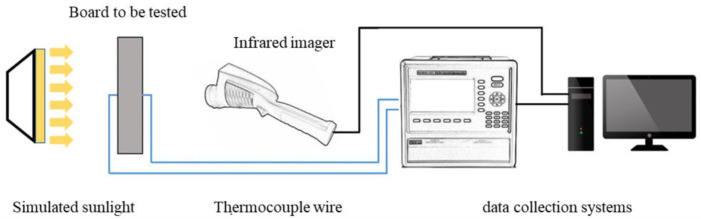
Test system for the single-side heating performance of concrete slabs.

**Figure 5:**
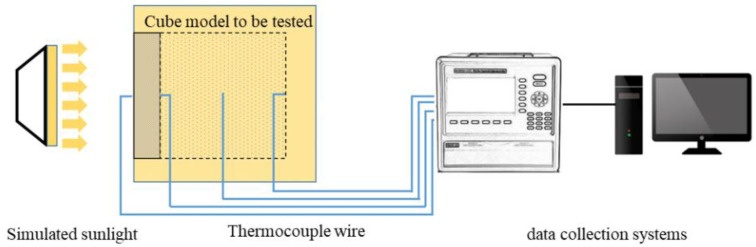
Test system for the thermal insulation performance of cube model.

(1) Mechanical properties: the loading rate of the cementitious composite sample is 1200 N/s, and three parallel tests are carried out for each group. According to GB/T50081-2019, the concrete compressive and splitting tensile performance tests are carried out. Maintain an ambient temperature of 20 ± 5 °C during the stress test.
(2) Early hydration temperature rise: Set all the raw materials at 20 °C for 24 h until constant temperature, prepare fresh phase change aggregate concrete, and pour into a 1 L cylindrical container with a temperature sensor vertically fixed in the center of the lid and connected to the SV3000 environmental monitoring system on the computer. After measurement, close the lid and make a temperature sensor insert to the core of fresh concrete, put the container as a whole into the incubator, and record the temperature every 1 min.
(3) Temperature regulating performance: paste thermocouple in the center of the two largest planes of basalt aggregate concrete slab and phase change aggregate concrete slab and use a high-power heating lamp to simulate sunlight irradiation on one side. The thermocouple is connected to the ToprietP700 multichannel data recorder and records the temperature at the center of the inner and outer surface of the concrete plate every 5 s. At the same time, RNO PC160 infrared imager produced by Meiketsu E-Commerce Co., Ltd., Shanghai, China, was used to take infrared images of the backlit surface of the concrete slab every 20 min. The test device for the concrete slab is shown in Figure 4.
(4) Thermal insulation performance: the phase change aggregate concrete slab and the basalt aggregate concrete slab were, respectively, combined with five polystyrene thermal insulation boards to make a cube model. All cracks in the model were filled with polyurethane foam sealant. High-power heating lamps were used to simulate sunlight irradiation. Thermocouples are installed in the center of the inner and outer sides of the concrete slab, the body center of the model, and the center of the polystyrene thermal insulation board on the bottom and connected with the TOPEIE TP700 multi-channel data recorder. The temperature is recorded every 5 s. The schematic diagram of the whole test device is shown in Figure 5.

## 3. Results and Discussions

### 3.1. Compressive Mechanical Properties

**Figure 6:**
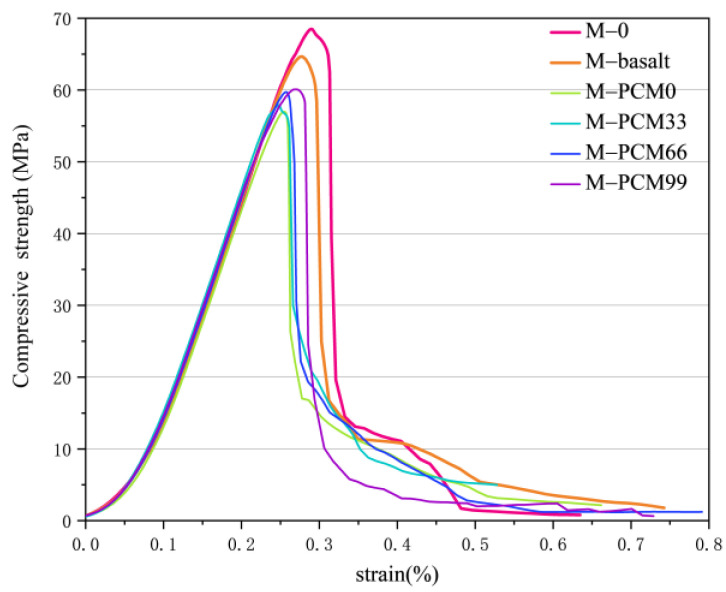
Stress and strain curve of cementitious composite samples under compression for 28 days.

The effects of aggregate type and paraffin filling on the compressive property of the phase change cementitious composite sample are shown in Figure 6. All curves are sharp, with a linear elastic rising stage, then rapidly dropping when it reaches failure, showing brittleness characteristics. The shapes of several curves are similar, indicating that different aggregate types and properties have little change in the failure process. It can be seen from the stress–strain curve that, compared with pure mortar M−0, the compressive strength of phase change cementitious composite containing aggregate decreases, whether basalt aggregate or phase change aggregate is added. It is important to consider the effect of the mass per unit volume of the sample on the strength. the 3D printed aggregate inside sample M−PCM0 was not filled with PCM and sample M−PCM99 was filled with PCM. By weighing the samples, it was found that the difference in weight between the two was very small, as the density of the filled PCM was very low. Such a difference in mass is negligible in relation to the total mass of the sample. Adding a small amount of aggregate to the cementitious composite and not accumulating dense, the limited amount of aggregate will not only fail to form the skeleton structure but also destroy the homogeneity of the mortar system and introduce a relatively weak interface. Therefore, adding a small amount of aggregate will lead to the decline of compressive strength.

**Figure 7:**
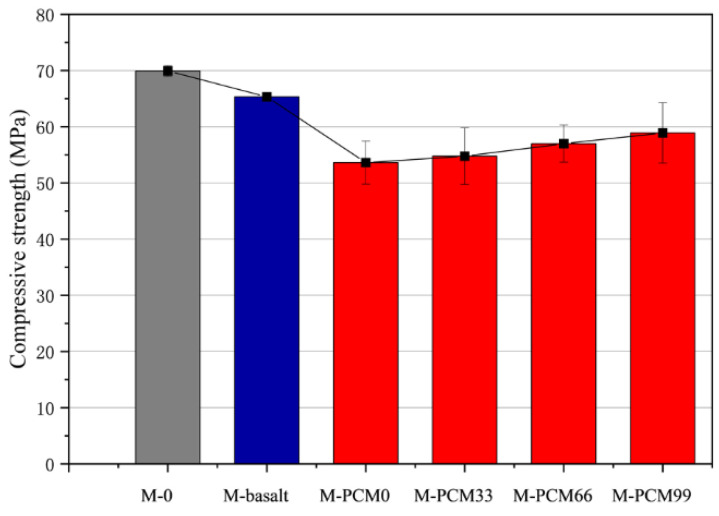
Compressive strength of cementitious composite samples for 28 days. (In the Figure the grey is pure mortar, the blue with basalt aggregate and the red with different phase change aggregates).

As shown in Figure 7, different aggregates in mortar lead to a different degree of compression strength decline. This is because the addition of aggregate introduces more defects. Adding basalt aggregate into mortar has a smaller effect on the decrease of compressive strength than adding phase change aggregate. This is due to the higher compressive strength of basalt aggregate. The cementitious composite strength will improve with the increase of paraffin volume fraction in the cavity of the phase change aggregate. In the hole of the phase change aggregate without PMC, the compressive strength of the mortar M−0 is 53.07 MPa, while the paraffin volume fraction in the cavity of the phase change aggregate is 99%, the compressive strength of cementitious composite M−PCM99 is 58.90 MPa, which increases by 9.9%. A similar pattern was derived by Sukontasukkul et al. [36], where the strength of mortar specimens showed an increase after increasing the proportion of light aggregates adsorbed with PEG. Phase change materials absorb energy during the phase change process, and their physical form is also altered accordingly. Thus, 3D printer aggregate filled with paraffin as PCM can improve the phase change cementitious composite compressive strength. It is associated with the physical properties of paraffin, which, encapsulated in the phase change aggregate, gradually transformed into a solid as temperature reduced, to enhance the mechanical properties of the whole structure of the aggregate.

**Figure 8:**
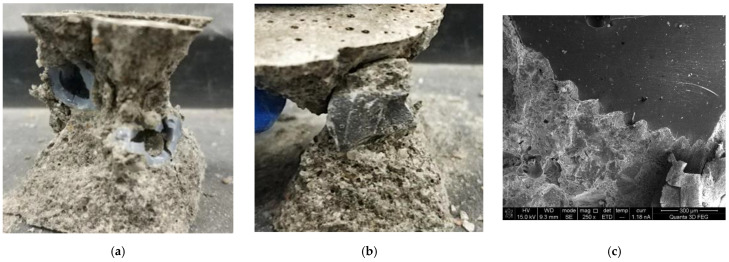
Damage condition of the cementitious composite samples. (**a**) Cementitious composite mixed with 3D−printed aggregate. (**b**) Cementitious composite mixed with basalt aggregate. (**c**) The interface between the phase change aggregate and the mortar matrix.

The damage to the cementitious composite under compression is shown in Figure 8. The failure modes of cementitious composite samples prepared by two different aggregates are different. The fracture of the phase change aggregate is shown in Figure 8a, while the basalt aggregate only has a large area of spalling with the surrounding cement matrix, as shown in Figure 8b. On the one hand, the compressive strength of the basalt aggregate is better than that of the phase change aggregate with cavities. On the other hand, the bond between the phase change aggregate and the cement mortar matrix is outstanding. This suitable bonding property is due to the uniform tentacle structure and serrated surface texture. As shown in Figure 8c, an SEM photograph of the interface between the phase change aggregate and the mortar matrix. This image shows the jagged surface of the phase change aggregate tightly bonded to the matrix.

**Figure 9:**
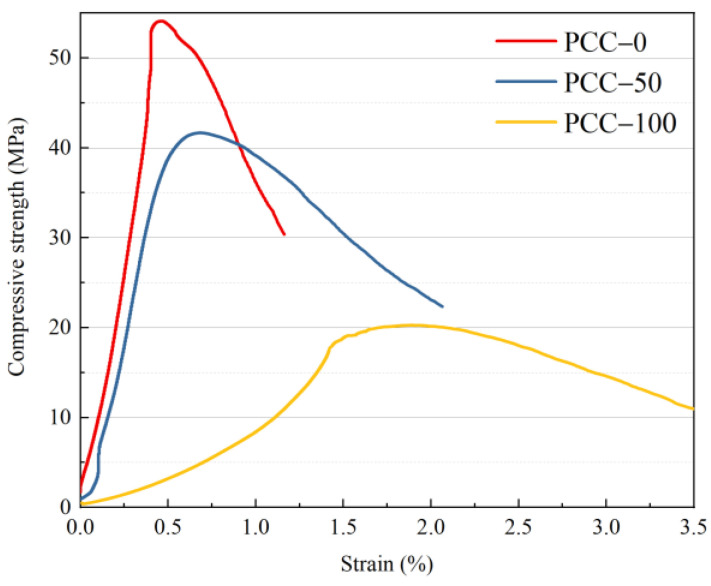
Stress–strain curve of concrete with different substitution amounts of phase change aggregates.

The compressive stress–strain curve of concrete prepared by phase change aggregate replacing basalt aggregate with volume ratios of 0%, 50%, and 100% are shown in Figure 9. The shapes of the three curves are entirely different. It can be seen that the curve of basalt aggregate concrete is sharp, and the stress rapidly declines when it reaches failure, showing brittleness characteristics. Under the 50% substitution rate, the trend of the curve of concrete PCC−50 is similar to that of basalt aggregate concrete PCC−0 in the rising stage, but after reaching the peak, the decreasing trend slows down. In the case of a 100% substitution rate, the curve of concrete PCC−100 has a gentle shape, and a plateau appears after reaching the maximum compressive strength, showing a high ductility. The strength of 3D printed phase change aggregate concrete decreased due to the structural stability problems posed by the cavities of the shell-core structure. The basalt aggregate concrete still exhibited brittle characteristics, with rapid stress decay occurring after maximum compressive strength was reached. When the two aggregates are used together, the stress–strain curve of the concrete under compression shows characteristics that lie between the two extremes, increasing the deformation capacity of the concrete while maintaining a high load-bearing capacity.

**Figure 10:**
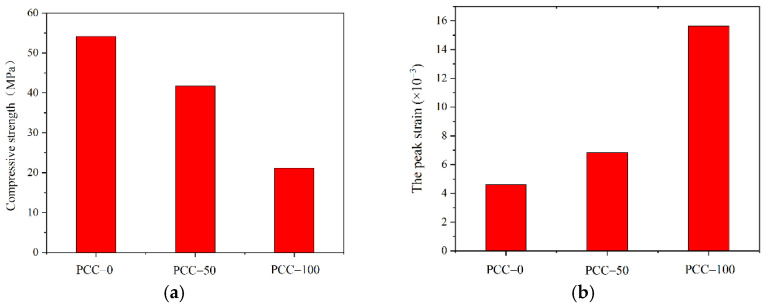
Compressive properties of phase change concrete. (**a**) Basalt aggregate. (**b**) Phase change aggregate.

**Figure 11:**
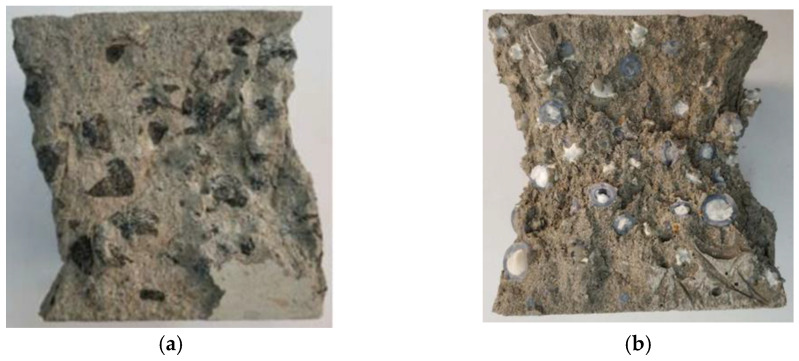
Damage condition of concrete with different aggregates. (**a**) Basalt aggregate. (**b**) Phase change aggregate.

The stress–strain curves of concrete prepared with two kinds of aggregate are further analyzed. As shown in Figure 10a, the compressive strength of PCC−100 reaches 21.18 MPa, while the compressive strength of PCC−50 is 41.68 MPa. The compressive strength of concrete decreases with the increase in phase change aggregate substitution rate. Similarly, Cao et al. [25] added PCM into concrete after encapsulation, which also caused a significant decrease in strength, up to 51%. However, it is worth mentioning that the phase change aggregate concrete is much lighter, with a specific strength of 11.48 kN·m/kg, close to lightweight aggregate structural concrete. As shown in Figure 11, The fracture of phase change aggregate during concrete failure indicates that the low strength of phase change aggregate leads to the decrease of concrete compressive strength, which is different from the poor interface performance of concrete caused by PCM. As shown in Figure 10b, the peak strain of concrete increases with the increase in substitution rate of phase change aggregate. The peak strain of basalt aggregate concrete PCC−0 is 4.61 × 10^−3^. The peak strain of concrete PCC−50 with a 50% phase change aggregate substitution rate is 6.85 × 10^−3^, 56.2% higher than that of basalt aggregate concrete. The peak strain of concrete PCC−100 with a 100% phase change aggregate substitution rate is 15.64 × 10^−3^, which increases by 70.5% compared to basalt aggregate concrete. The incorporation of phase change aggregate makes the concrete show good ductility. The compressive strength decreases while the ductility increases because the cavity inside the phase change aggregate reduces the mechanical properties and improves the deformation property of aggregate. Moreover, when the partial phase change aggregate replaces basalt aggregate, they disperse each other, which significantly weakens the function of the “self-locking” tentacle structure added to the aggregate design.

**Figure 12:**
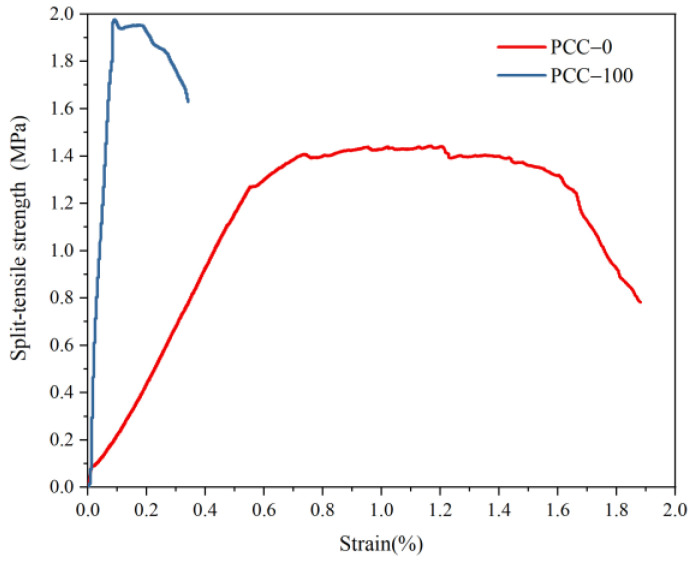
Effect of 3D-printed phase change aggregate on splitting tensile properties of concrete.

The splitting tensile properties of concrete prepared with two kinds of aggregate are shown in Figure 12. The basalt aggregate concrete reaches its maximum splitting tensile strength at the end of the elastic stage and then experiences a short yield stage and cracks rapidly. After the elastic stage of phase change aggregate concrete, the failure of the structure occurs after a long period of the yield stage, and the maximum splitting strength is in the middle of the yield platform. The splitting strength of PCM concrete is 1.45 MPa, and that of basalt aggregate concrete is 1.97 MPa, which is close, but the peak strain is nearly 13 times that of basalt aggregate concrete. The resin phase change aggregate is used to construct the skeleton structure, which can effectively improve the ductility of concrete when it is subjected to tension. The appearance of a large platform proves that energy is effectively absorbed by the shedding of the tentacle structure on the surface of bionic aggregate and the rupture of the shell core structure of aggregate.

### 3.2. Influence of Hydration Temperature Rise

The hydration reaction of cement is exothermic, and the poor thermal conductivity of concrete is easy to produce temperature differences inside the concrete, which leads to cracking of concrete due to temperature stress. Incorporating phase change materials in concrete reduces the early hydration temperature rise rate. Kim et al. [35], pointed out that PCM plays an essential role in reducing the thermal stress of concrete. Small temperature fluctuations were observed in the early hydration process of concrete using PCM.

**Figure 13:**
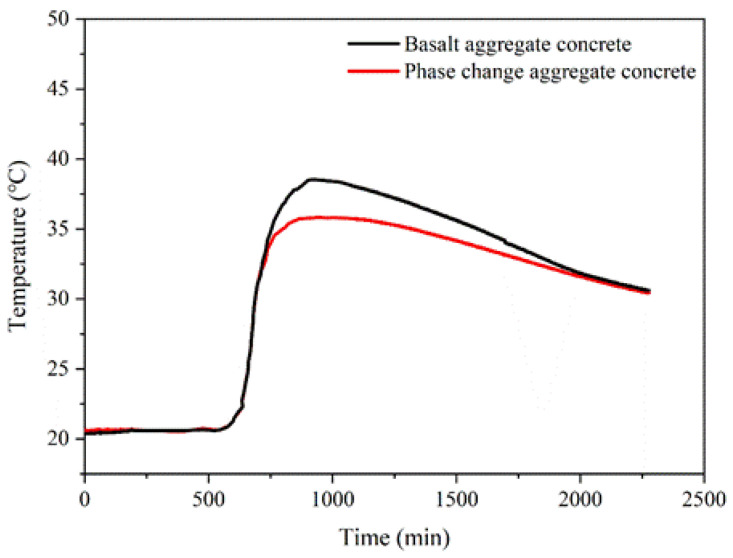
Early hydration temperature rises the curve of concrete.

The inhibition effect of printed phase change aggregate on early temperature rise of concrete is shown in Figure 13. From the beginning of measurement to 525 min, the temperature rises rapidly, and the hydration reaction rate begins to accelerate. The exothermic rate reached the maximum at about 680 min. About 700 min later, the effect of the phase change aggregate began to show. The paraffin phase change process absorbs a lot of heat and slows the temperature rise inside the concrete. The peak temperature of early hydration temperature rises of phase change aggregate concrete is lower, and the curve is gentler. At the peak, the internal temperature of basalt aggregate concrete is 38.56 °C, and the internal temperature of phase change aggregate concrete is 35.82 °C. The heat released by the hydration reaction was absorbed by paraffin in the cavity of phase change aggregate through the phase change process, which reduced the internal peak temperature of concrete by 7.1%. The use of phase change aggregate made the exothermic stage of concrete gentler, and the inner temperature curve of concrete became smoother. It can be seen that the phase change aggregate effectively inhibits the early hydration temperature rise of concrete.

### 3.3. Analysis of Temperature Regulation Performance

**Figure 14:**
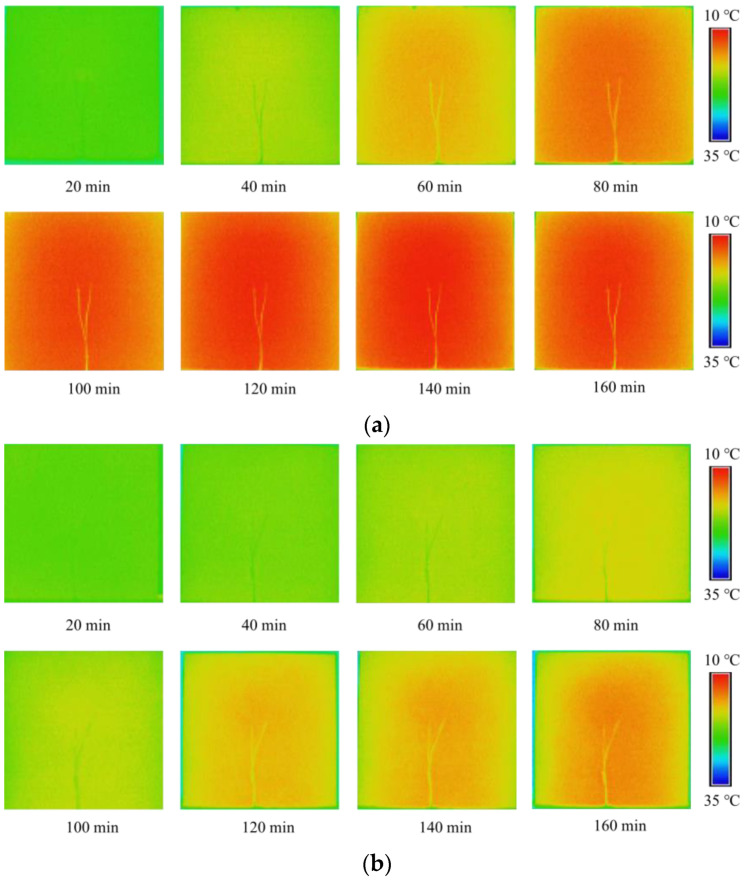
Temperature distribution of two kinds of aggregate concrete slabs. (**a**) Basalt aggregate concrete slab. (**b**) Phase change aggregate concrete slab.

The temperature distribution of the shady surface of concrete slabs with the two kinds of aggregate varies with illumination time, as shown in Figure 14. The two thin lines in the center of the picture are thermocouple lines. The color change in the image reflects the difference in surface temperature. The lower temperature limit is set to 10 °C, and the display color is blue, while the upper limit is set to 35 °C and the display color is red. The temperature of the shady surface of the basalt aggregate concrete slab changes rapidly, and the surface temperature distribution is more uniform. The temperature of the shady surface of the phase change aggregate concrete slabs varies slowly, showing lower temperatures than that of the basalt concrete slabs through the same time. The distribution of the red regions showed slight inhomogeneity in the infrared imaging images at 120 min and 140 min. The slowing down of the temperature rise rate and the inhomogeneity of temperature distribution shows the effect of temperature regulation by the phase transition process of paraffin in the phase change aggregate. The difference in temperature distribution proves that phase change aggregates are effective for energy storage and can be applied to energy-efficient buildings. Pop et al. [37] calculated the energy efficiency of a virtual office building with the application of phase change materials and concluded that PCM thermal storage walls effectively saved electricity supply.

**Figure 15:**
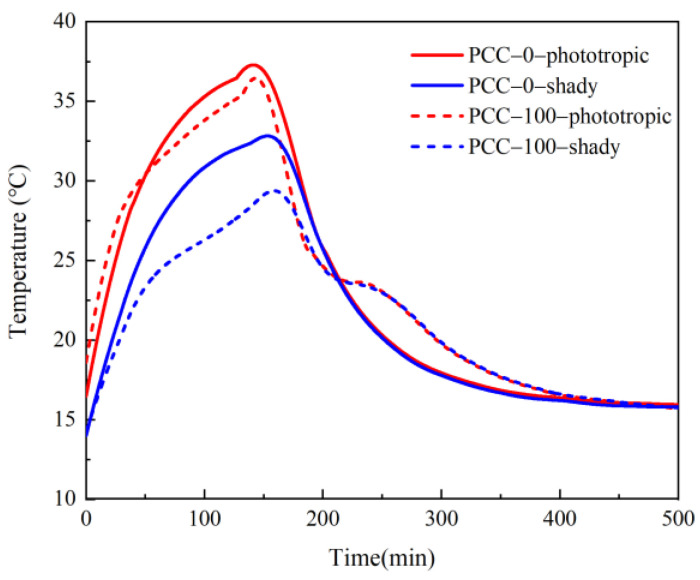
The curve of the center temperature of the inner and outer surfaces of concrete slabs.

The temperature of the concrete slab on the phototropic surface and the shady surface changing with time is plotted, as shown in Figure 15. The lighting starts at 0 min and ends at 160 min. The period is the heating stage, and the temperature presents an upward trend. The heating rate of basalt aggregate concrete slab on the phototropic surface and the shady surface both decreases gradually. The heating rate reaches its lowest in 160 min, and the temperature curve also tends to be flat. The temperature change of phase change aggregate concrete slab is divided into two stages. In the first stage, similar to basalt aggregate concrete, the temperature rise rate decreases gradually. In the second stage, the rising rate of the center temperature of the two surfaces tends to increase. At 99 min, the maximum temperature difference on the shady surface center between phase change aggregate concrete and basalt aggregate concrete was 4.8 °C. At 107 min, the maximum temperature difference on the phototropic surface center between phase change aggregate concrete and basalt aggregate concrete was 1.9 °C. After the illumination stops, 160 min to 500 min is the cooling stage, and the temperature shows a downward trend. At this time, the basalt aggregate concrete slab temperature also decreases gradually. After 420 min, the temperature of the basalt aggregate concrete slab approaches room temperature, and the curve begins to be parallel to the x-axis. The temperature change of phase change aggregate concrete slab is also obviously divided into two stages. The first stage is similar to the basalt aggregate concrete slab, and the temperature decline rate gradually decreases. The temperature almost stops falling in the second stage, and the curve enters a platform stage. After the short platforming period, the temperature of the phase change aggregate concrete slab began to decline at an accelerated pace. Around 280 min, the temperature decline rate reached its maximum and began to decrease gradually, and the curve was almost parallel to the x-axis after 420 min. PCM makes a difference in the pattern of temperature change in concrete as a direct result of PCM absorbing heat without increasing the surrounding temperature. A similar phenomenon was reported by Pongsopha et al. [38]. The phase transition of paraffin makes the surface temperature of phase change aggregate concrete higher than that of basalt aggregate concrete for a while. For the phototropic surface, the temperature difference between the phase change aggregate concrete and basalt aggregate concrete reaches a maximum of 3.0 °C at 260 min. For the shady surface, the temperature difference between the phase change aggregate concrete and basalt aggregate concrete reaches a maximum of 3.2 °C at 267 min.

The temperature change of phase change aggregate concrete presents relatively complex fluctuations because the paraffin inside the aggregate has phase change in both the heating and cooling stages, which affects the heat transfer in the direction of the temperature gradient. The addition of PCM leads to the difference in the temperature variation pattern of concrete. During the heating process, paraffin stores heat through a phase change, resulting in a slow temperature rise. At the same time, the temperature of phase change concrete is always lower than that of basalt concrete, and the final temperature is also lower. In the cooling process, it is also due to the heat released by the phase change of paraffin, which delays the temperature reduction process of the phase change concrete slab.

**Figure 16:**
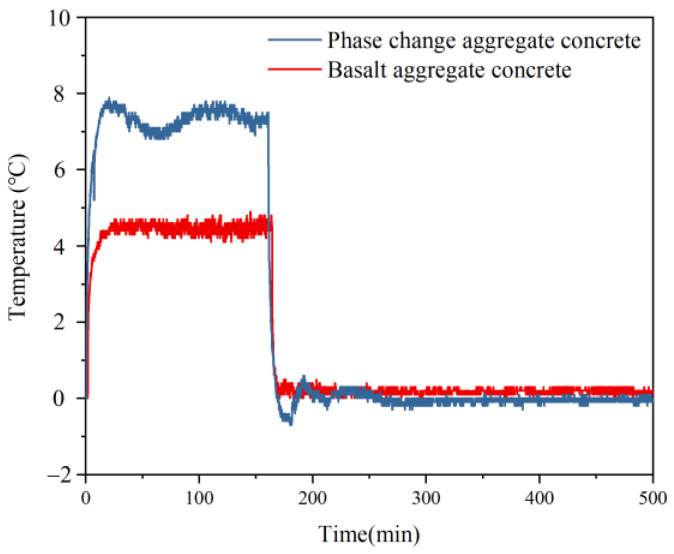
The temperature difference between inner and outer surfaces of concrete slabs.

The phase change process also significantly influences the temperature difference between the phototropic and shady surfaces of concrete, as shown in Figure 16. The temperature difference between the two sides of the basalt aggregate concrete has violent fluctuations at the beginning and the end of illumination. It rises and decreases rapidly and then enters the platform stage. The temperature difference only fluctuates within a tiny range when the external environment does not change. When exposed to lighting for 20 min, the temperature difference between the phototropic surfaces and shady surfaces of the basalt aggregate concrete is 4.9 °C. The temperature difference between the phototropic and shady surfaces of phase change aggregate concrete fluctuates wildly. Whether in the process of heating up or cooling down, after the temperature difference between the phototropic surfaces and shady surfaces of the phase change aggregate concrete reaches the maximum, the vibration with gradually decreasing amplitude is carried out near the stable temperature difference line. It may be that the time of paraffin phase change in phase change aggregate is not synchronous, resulting in uneven temperature distribution in concrete. In the heating stage, the temperature difference first increases rapidly to 7.9 °C, which is also the maximum temperature difference in this stage. Then the temperature difference fluctuates up and down, and the final stable temperature difference is near 7.3 °C. After stopping the illumination, the temperature difference first decreases rapidly to 0.7 °C, which is also the minimum temperature difference at this stage. Then, the temperature difference also fluctuates up and down and finally stabilizes at 0 °C.

### 3.4. Thermal Insulation Performance Analysis

**Figure 17:**
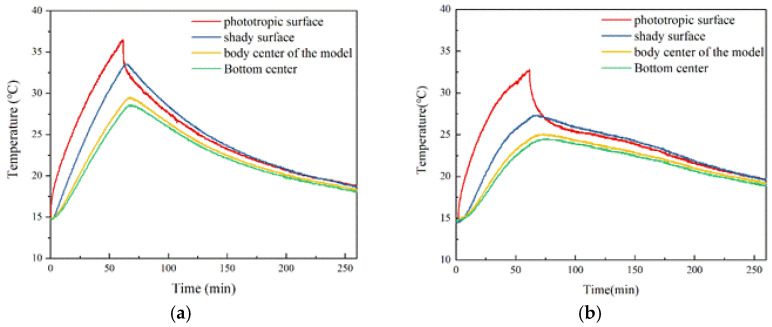
Temperature changes at each point of the cube model. (**a**) Basalt aggregate concrete cube model. (**b**) Phase change aggregate concrete cube model.

Temperature changes at each point in the cube model are shown in Figure 17. The temperature change of each measuring point in the cube model of basalt aggregate concrete is relatively rapid, whether in the heating or cooling stage. After starting lighting, the rate of temperature increases at all points, maintaining a decreasing trend until the temperature reached its peak. After stopping the light at 61 min, the rate of temperature decline at each point also showed a relatively uniform decreasing trend, and the temperature curve tended to be flat with the increase of time. Different from the single concrete slab, the temperature of the shady surface of the concrete slab is always higher than the temperature of the phototropic surface during the shady surface temperature drops from the peak. The temperature change curve of the cube model prepared with phase change aggregate concrete is also similar to that of a single concrete slab. In the heating stage, the temperature rise rate decreases significantly due to the phase transition. With the end of the phase transition process, the temperature rise rate begins to increase again until the end of the irradiation. In the 61st minute, the irradiation stopped, the temperature started to drop at each measuring point, and the temperature drop rate of phase change aggregate concrete slab significantly reduced due to the liquid–solid phase transition. At the end of the phase transformation process of the phase change aggregate inside the concrete slab, the decreasing rate of temperature begins to increase.

**Figure 18:**
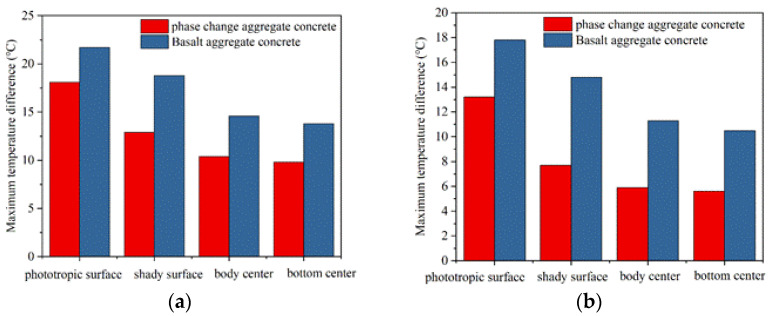
A maximum temperature difference of cube model. (**a**) Heating stage. (**b**) Cooling stage.

The maximum temperature difference at each temperature measuring point in the heating and cooling stages is shown in Figure 18. In the heating stage, the maximum temperature difference at each measuring point of the basalt concrete slab cube model is 21.7 °C, 18.8 °C, 14.6 °C, and 13.8 °C, respectively. The corresponding values of the phase change aggregate concrete slab cube model are reduced by 16.6%, 31.4%, 28.8%, and 29.0%, respectively. In the heating stage, Figure 17 also shows that the temperature of each measuring point in the cube model prepared with basalt aggregate concrete is always higher than that of the corresponding measuring point in the cube model prepared with phase change aggregate concrete at the same time. In the cooling stage, the maximum temperature difference of each measurement point of the basalt concrete slab cube model is 17.8 °C, 14.8 °C, 11.3 °C, and 10.5 °C, respectively. The corresponding temperature difference at each point is reduced by 25.8%, 48.0%, 47.8%, and 46.7%, respectively. After the 135th minute, Figure 17 also shows that the temperature of each measuring point in the cube model prepared with basalt aggregate concrete is always higher than that of the corresponding measuring point in the cube model prepared with phase change aggregate concrete at any same time. Comparing the two kinds of cube model, we can find that the temperature rise rate and temperature drop rate of the cube model prepared with phase change aggregate concrete are relatively slower. The maximum temperature difference of each temperature measuring point in the cubic model of phase change aggregate concrete is relatively lower. The above results show that the thermal insulation performance of the phase change aggregate concrete is better. In the process of heating and cooling, phase change aggregate plays a vital role in the storage and re-release of heat.

## 4. Conclusions

In this work, 3D printing technology and macro encapsulation prepared a phase change aggregate containing paraffin. The shell of phase change aggregate is formed by curing with high-strength resin. The surface of the shell has a tentacle structure to enhance mechanical properties. The cavity inside the phase change aggregate is filled with molten liquid paraffin, and the filling hole is sealed with epoxy resin glue. The mechanical and thermal properties of the phase change aggregate concrete were studied. The following conclusions were drawn:

(1) Increasing phase change materials in the cavity of phase change aggregate will improve its mechanical properties. Compared with the phase change aggregate without paraffin adsorption, the compressive strength of the phase change cementitious composite with the internal cavity filled with paraffin increased by 9.9%.
(2) The compressive strength of phase change aggregate concrete is 21.18 MPa, and the specific strength is 11.48 kN·m/kg. The concrete experiences ductile failure with the addition of phase change aggregate of 3D printing. When the replacement rate is 50% and 100%, the compressive peak strain of concrete increases by 48.6% and 239.3%, respectively. The splitting tensile strength was 1.45 MPa. The peak strain is 11.69 × 10^−3^, nearly 13 times that of basalt aggregate concrete.
(3) The addition of phase change aggregates reduces the hydration heating rate while improving thermal regulating and thermal insulation properties. The phase change aggregate reduced concrete’s early peak hydration temperature by 7.1%. For concrete slabs, phase change aggregates can effectively delay heat transfer. In the thermal insulation experiment, phase change aggregate can effectively store thermal energy, maintain the internal temperature of the model, and improve the thermal insulation performance of phase change concrete.

## Figures and Tables

**Table 1:** Gradation of phase change aggregate.

D (mm)	2.36	4.75	7.5	9.5	12.5	16	17.5
Wt (%)	4.30	7.79	17.46	29.51	25.50	8.87	6.57

**Table 2:** Chemical composition of cement.

Chemical Composition	CaO	SiO_2_	Al_2_O_3_	Fe_2_O_3_	MgO	SO_3_
content (%)	63.62	20.13	4.71	3.35	1.26	2.85

**Table 3:** Chemical composition of fly ash.

Chemical Composition	CaO	SiO_2_	Al_2_O_3_	Fe_2_O_3_	MgO	SO_3_	Na_2_O	TiO_2_
content (%)	7.62	48.3	28.7	7.2	1.29	0.95	0.86	1.46

**Table 4:** Mixture proportions of phase change cementitious composite.

	Aggregate (Number)	PCMs Filling Rate	Cement (kg)	Fly Ash (kg)	Sand (kg)	Water (kg)	Superplasticizer (kg)
M−0	0	0	355.88	100.35	798.41	182.24	0.57
M−basalt	6	0	355.88	100.35	798.41	182.24	0.57
M−PCM0	6	0	355.88	100.35	798.41	182.24	0.57
M−PCM33	6	33%	355.88	100.35	798.41	182.24	0.57
M−PCM66	6	66%	355.88	100.35	798.41	182.24	0.57
M−PCM99	6	99%	355.88	100.35	798.41	182.24	0.57

**Table 5:** Mixture proportions of phase change concrete.

	Basalt Aggregate (kg)	Phase Change Aggregate (kg)	Cement (kg)	Fly Ash (kg)	Sand (kg)	Water (kg)	Superplasticizer (kg)
PCC−0	1016.16	0	355.88	100.35	798.41	182.24	0.57
PCC−50	508.08	182.25	355.88	100.35	798.41	182.24	0.57
PCC−100	0	364.51	355.88	100.35	798.41	182.24	0.57

## Data Availability

The data presented in this study are available on request from the corresponding author. The data are not publicly available due to privacy.
